# Exploring Carbon Monoxide and Carbon Dioxide Adsorption on (5,5) Aluminum Nitride Nanotubes for Enhanced Sensor Applications: A DFT Study

**DOI:** 10.3390/molecules29030557

**Published:** 2024-01-23

**Authors:** Nafiu Suleiman, Vitus Atanga Apalangya, Bismark Mensah, Kwabena Kan-Dapaah, Abu Yaya

**Affiliations:** 1 Department of Materials Science and Engineering, College of Basic and Applied Science (CBAS), University of Ghana, Accra P.O. Box LG 77, Ghana; nsuleiman005@st.ug.edu.gh (N.S.); bismarkmensah@ug.edu.gh (B.M.); 2 Department of Food Process Engineering, College of Basic and Applied Science (CBAS), University of Ghana, Accra P.O. Box LG 77, Ghana; vapalangya@ug.edu.gh; 3 Department of Biomedical Engineering, College of Basic and Applied Science (CBAS), University of Ghana, Accra P.O. Box LG 77, Ghana; kkan-dapaah@ug.edu.gh

**Keywords:** CO, CO_2_, density functional theory (DFT), nanotubes, HOMO–LUMO orbitals, sensors

## Abstract

This study examined the sensitivity of single-walled (5,5) aluminium nitride nanotubes ((5,5) AlNNTs) to carbon monoxide (CO) and carbon dioxide (CO_2_) gas molecules by performing DFT calculations using a hybrid functional, specifically, B3LYP (Becke’s three-parameter, Lee-Yang-Parr) exchange-correlation functional at a 6–31G* basis set. This research investigates the adsorption behavior of CO_2_ and CO molecules on pristine and silicon-doped aluminum nitride nanotubes (AlNNTs) and examines their implications for sensor applications. The study assesses each system’s adsorption energy, sensing potential, and recovery time to gain insights into their binding strength and practical viability. For CO_2_ adsorption on (5,5) AlNNT, significant adsorption energy of −24.36 kcal/mol was observed, indicating a strong binding to the nanotube surface, with a sensing potential of 8.95%. However, the slow recovery time of approximately 4.964 days may limit its real-time application. Si-(5,5) AlNNT exhibited a CO_2_ adsorption energy of −19.69 kcal/mol, a sensing potential of 5.40%, and a relatively short recovery time of approximately 2.978 min, making it a promising candidate for CO_2_ sensing. CO adsorption on (5,5) AlNNT showed an adsorption energy of −25.20 kcal/mol, a sensing potential of 9.095%, but a longer recovery time of approximately 20.130 days. Si-(5,5) AlNNT displayed a high CO adsorption energy of −20.78 kcal/mol, a sensing potential of 4.29%, and a recovery time of approximately 18.320 min. These findings provide insights into the adsorption characteristics of carbon molecules on AlNNTs, highlighting their potential for CO_2_ and CO sensing applications.

## 1. Introduction

Carbon monoxide (CO) and carbon dioxide (CO_2_) are two important gases that significantly impact human health and the environment. Carbon monoxide (CO) is a tasteless gas that has no smell [1,2]. It is produced from the incomplete combustion of carbon-containing materials such as fuel, combustible gas, and wood. Major sources include releases from automobile engines, power plants, wildfires, and incinerators [2]. Similarly, photochemical processes in the atmosphere can cause methane and nonmethane hydrocarbons, other unstable natural hydrocarbons in the air, natural atoms in surface waters, and soils to all interact and form CO. CO is a toxic gas that is produced by the burning of fossil fuels. It can cause headaches, nausea, and dizziness, and in high concentrations, it can be fatal [1,2,3]. CO_2_, on the other hand, is a greenhouse gas that is produced by human activities such as the burning of fossil fuels. It contributes to climate change by trapping heat in the atmosphere [1,2,3,4,5]. According to the World Health Organization (WHO), exposure to high levels of carbon monoxide can lead to unconsciousness and death. In the United States, the Environmental Protection Agency (EPA) estimates that more than 400 Americans die each year from accidental nonfire-related CO poisoning [6,7,8,9,10,11]. CO_2_ is also a major concern. According to the Intergovernmental Panel on Climate Change (IPCC), human activities are the main driver of climate change, and fossil fuel burning is the largest contributor to CO_2_ emissions [8,12,13,14,15,16,17,18,19]. The concentration of CO_2_ in the atmosphere has risen from around 280 parts per million (ppm) in the pre-industrial era to over 400 ppm in recent times [20,21,22,23,24]. This has led to an increase in global temperature and more extreme weather events such as heat waves, droughts, and storms [25,26,27,28,29,30,31,32,33].

In addition to the impact on human health and the environment, CO and CO_2_ can also have significant economic consequences. For example, the costs of treating illnesses related to air pollution and the costs of adapting to climate change are expected to increase [34,35,36,37,38,39].

AlNNTs have emerged as a unique and highly promising class of nanomaterials that have garnered significant attention in recent years due to their exceptional properties and wide range of potential applications. These nanotubes possess remarkable characteristics, including high thermal conductivity, excellent mechanical strength, and outstanding chemical stability [40]. The high thermal conductivity of AlNNTs makes them particularly suitable for thermal management applications. With their ability to efficiently conduct heat, AlNNTs can be utilized to design and develop thermal management systems. This is particularly important in electronic devices and integrated circuits, where effective heat dissipation is crucial for enhancing performance and reliability [41].

Furthermore, the excellent mechanical strength of AlNNTs opens up opportunities for their use in structural materials and composites. By incorporating AlNNTs into various matrices, such as polymers or metals, the resulting composites can exhibit improved mechanical properties, including enhanced strength and durability. This makes AlNNTs promising for aerospace, automotive, and construction applications, where lightweight yet strong materials are highly sought after [40].

The exceptional chemical stability of AlNNTs allows them to withstand harsh environments, making them suitable for applications in corrosive or chemically aggressive settings. AlNNTs can be employed in sensors for detecting gases, liquids, or even biological analytes. Leveraging their chemical stability, AlNNT-based sensors have the potential to enhance sensitivity, selectivity, and response time, leading to advancements in environmental monitoring, medical diagnostics, and industrial sensing [42,43,44,45]. Energy storage is another area where AlNNTs show great promise. Their unique properties can be leveraged to improve energy storage devices’ performance and efficiency, such as in batteries and supercapacitors. AlNNTs hold the potential to increase energy density, thus improving cycling stability and reducing charging time, which are crucial factors in advancing energy storage technologies [40,42,44]. 

To synthesize AlNNTs, various methods have been explored. Chemical vapor deposition, chemical etching, and electrochemical techniques are among the commonly employed approaches. The choice of synthesis method plays a critical role in determining the structure, morphology, and properties of the resulting nanotubes. Researchers are actively investigating and optimizing these synthesis techniques to achieve AlNNTs with the desired characteristics for specific applications [40,41,42,43,44,45]. 

The field of AlNNT research is rapidly expanding, with numerous studies focused on the synthesis, characterization, and potential applications of these materials. Scientists are striving to gain a deeper understanding of the fundamental properties and behaviors of AlNNTs and to explore novel applications across various industries. The continuous efforts in this field hold promise for further advancements and breakthroughs that can unlock the full potential of AlNNTs as a versatile and high-performance nanomaterial [44,45,46,47].

In recent studies, the adsorption behavior of O_2_ molecules on silicon-doped graphene (C_53_H_18_Si) has been explored. Kuzmin and Shainyan (2020) discovered that the adsorption of O_2_ on silicon proceeded without any barriers, resulting in the formation of exclusively atop intermediate. The adsorption energy for this process was found to be −2.40 eV. Interestingly, the barrier to dissociation of O_2_ adsorbed on Si-doped graphene was approximately 16 times lower compared to pristine graphene [48].

In a separate investigation, Zhao et al. (2012) focused on graphene doped with a single Si atom, achieved by replacing one carbon atom with a silicon atom. The adsorption process was studied specifically for the doped site. Notably, the only observed adsorption occurred between the oxygen atom of the H_2_O molecule and the silicon atom. It should be noted that the H_2_O molecule did not adsorb onto the silicon atom from its hydrogen atoms or other adjacent positions [49]. Milad et al. (2020) also reported an adsorption energy of −29.92 kcal/mol, which is approximately −1.297 eV, for the interaction between H_2_O molecules and Si-doped graphene. This finding aligns with the previous study conducted by Zhao et al. (2012) [50]. Overall, the studies on silicon-doped graphene demonstrate the influence of silicon doping on gas adsorption properties. This knowledge can be extended to other silicon-doped materials, including silicon-doped aluminum nitride nanotubes, to design and optimize gas sensors with improved performance for sensing CO and CO_2_ gases in various environments and applications. The ability to enhance gas sensing properties through silicon doping makes it a promising approach to developing advanced gas sensors with practical applications in environmental monitoring, industrial safety, and more.

Doped AlNNTs represent a novel and highly intriguing class of nanomaterials that have gained considerable attention in recent years. These doped nanotubes exhibit unique properties and offer promising potential applications, particularly in the field of sensing [50,51,52,53,54,55]. Impurity doping of AlNNTs, involving the introduction of elements such as copper, carbon, and titanium, has been found to significantly enhance their sensing capabilities towards specific chemicals, such as formaldehyde (HCOH) and ammonia (NH_3_) [53,55]. The addition of impurities brings about changes in the electrical conductivity and bandgap of AlNNTs, which can be exploited for sensing purposes. For instance, doping AlNNTs with copper can transform them into p-type semiconductors, thereby improving their sensing properties. This modification leads to increased sensitivity and selectivity, making them more suitable for detecting and sensing specific analytes [52,53,54,55,56]. 

The introduction of impurities in AlNNTs brings about several benefits for sensing applications. Firstly, it can alter the electronic structure of the nanotubes, influencing their conductivity and bandgap. This modification enables tailored responses to target analytes, enhancing the sensitivity and selectivity of the sensing material. By precisely controlling the type and concentration of the dopants, the sensing performance of AlNNTs can be optimized for specific chemical species [52,55]. Secondly, the introduction of impurities can induce localized defect states within the bandgap of AlNNTs. These localized states serve as active sites for chemical interactions, allowing for enhanced adsorption and reactivity toward target analytes. This feature contributes to the improved sensitivity and selectivity of doped AlNNTs in sensing applications [50,51,52,53,54,55]. The unique combination of impurity doping and the inherent properties of AlNNTs, such as high thermal conductivity and mechanical strength, further enhances their sensing capabilities. The resulting doped AlNNTs offer not only high-performance sensing platforms but also potential advancements in areas such as gas sensing, environmental monitoring, and biomedical applications. The exploration of doped AlNNTs as sensing materials represents a rapidly growing research field, with numerous studies focused on understanding the synthesis, characterization, and sensing performance of these materials. The ability to tailor the properties of AlNNTs through impurity doping opens up exciting possibilities for developing next-generation sensing devices with improved performance and expanded application domains.

This paper aimed to evaluate the performance of pristine AlNNT and Si-doped AlNNT as CO and CO_2_ gas sensors using DFT calculations. The electrical properties, as well as its interactions of CO and CO_2_ with armchair AlNNTs (5,5) and Si-doped AlNNTs (5,5), were thoroughly explored.

## 2. Results and Discussion

### 2.1. Pristine Aluminum Nitride Nanotube

**Figure 1:**
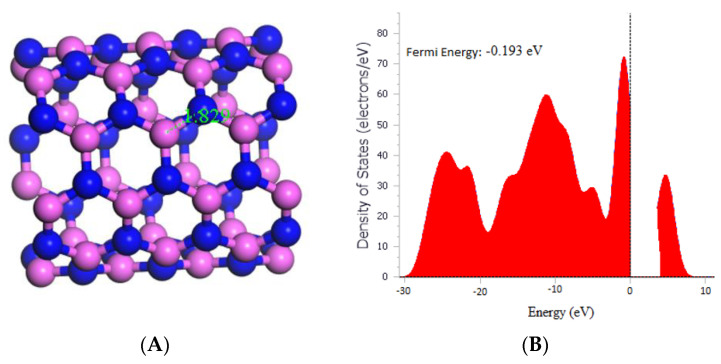
(**A**) Geometry optimized structure of the (5,5) AlNNT with the bond length measured in Å, and (**B**) Density of states for pristine (5,5) AlNNT.

Based on the results, it was observed that the bond length between Al and N in the (5,5) AlNNT was 1.829 Å, as depicted in Figure 1A, which matches a previous report by Mahdavifar et al. in 2013. Moreover, the band gap (Eg) was measured to be 4.090 eV, indicating that the material holds promise as a semiconductor. Furthermore, Figure 1B revealed that the fermi energy was −0.180 eV. In Table 1, the chemical potential (µ) was determined to be −4.265 eV, which implies that the material was stable. The global hardness (η) was calculated to be 2.045 eV, and the electrophilicity index (ω) was found to be 4.447 eV, which suggested that the material has a high electron affinity.

### 2.2. Silicon-Doped (5,5) Aluminum Nitride Nanotube [Si-(5,5) AlNNT]

**Figure 2:**
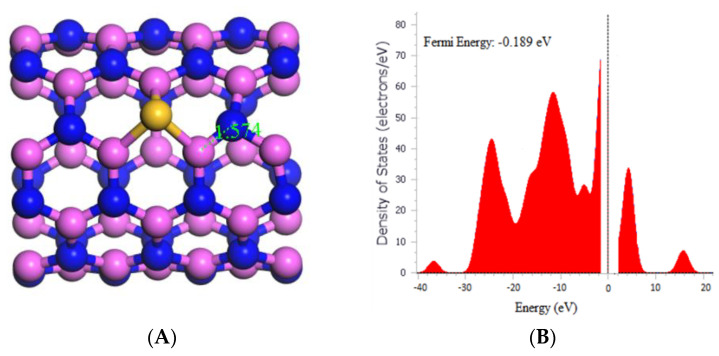
(**A**) Geometry optimized structure of Si-(5,5) AlNNT with the bond length measured in Å, and (**B**) density of states for Si-(5,5) AlNNT.

The data presented in Figure 2A shows that the bond length between Aluminum and Nitrogen in the AlNNT decreased from 1.829 to 1.574 Å after doping with silicon, indicating a significant impact on the electronic structure of the material. The calculated band gap (Eg) obtained from Table 1 also decreased from 4.090 to 3.725 eV after silicon doping. This reduction in Eg suggests the creation of new electronic states in the AlNNT, which could alter its electrical conductivity and optical properties. Hence, the decrease in Eg indicates an increase in the conductivity of AlNNT after doping. The fermi energy of the AlNNT also changed from −0.193 to −0.189 eV after doping, as observed in Figure 2B. Furthermore, the silicon doping also affected the chemical potential (µ), global hardness (η), and electrophilicity index (ω) of the AlNNT, as shown in Table 1. The chemical potential decreased from −4.265 to −4.131 eV after doping, indicating the presence of new chemical states introduced in the AlNNT. The global hardness decreased from 2.045 to 1.863 eV, indicating that the AlNNT could have become less resistant to deformation after doping. In contrast, the electrophilicity index increased from 4.447 to 4.580 eV, suggesting that the AlNNT could have become more susceptible to chemical reactions after doping.

### 2.3. CO_2_ Adsorbed on (5,5) AlNNT [(5,5) AlNNT/CO_2_]

**Figure 3:**
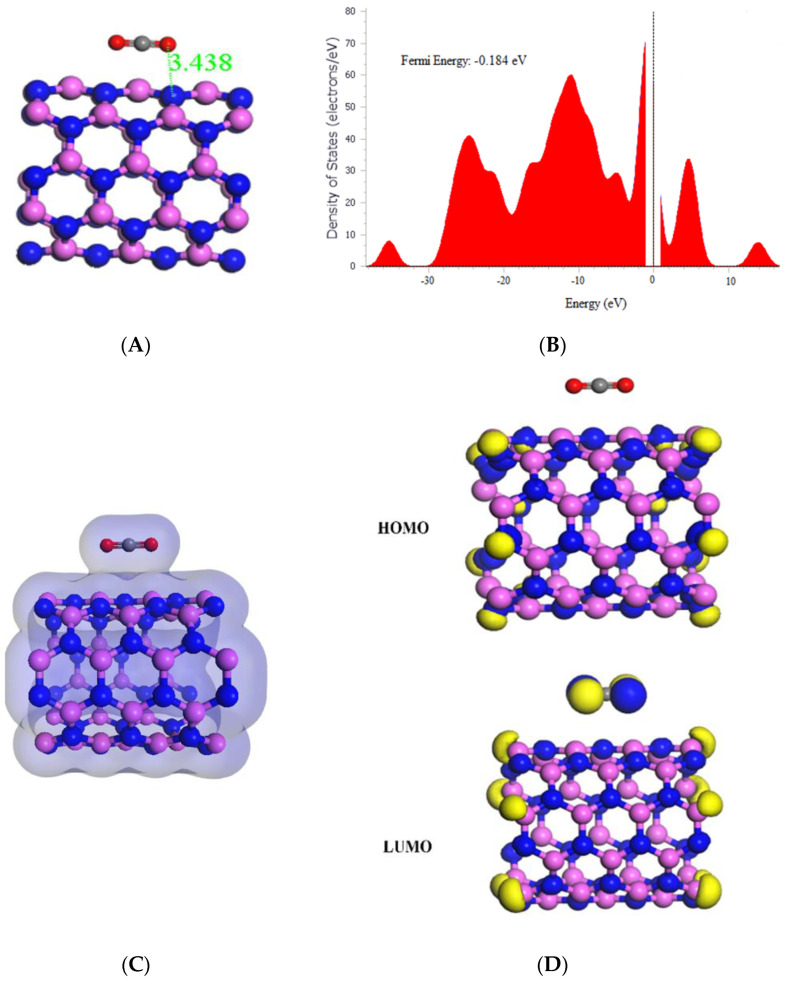
(**A**) Geometry optimized structure with the adsorption distance measured in Å, (**B**) density of states, (**C**) charge density, and (**D**) frontier HOMO and LUMO orbitals for the (5,5) AlNNT and its interaction with CO_2_.

This study investigated the interaction between CO_2_ and AlNNT (5,5) through adsorption, which resulted in the significant adsorption energy of −24.36 kcal/mol, indicating a strong binding between the CO_2_ molecule and (5,5) AlNNT surface, as shown in Table 1. The electronic structure of AlNNT (5,5) was modified by CO_2_ adsorption, as seen in the change of HOMO and LUMO values and a decrease in the band gap from 4.090 eV to 3.724 eV. The sensing potential of 8.95%, demonstrated in Table 1, suggests that (5,5) AlNNT has the potential to be used as a CO_2_ sensor; however, the slow recovery time of 428,899.920 s, which is approximately 4.964 days after CO_2_ adsorption, may limit its practical application. The chemical potential of −4.164 eV, global hardness of 1.862 eV, and electrophilicity index of 4.656 eV suggest that the material is less stable and softer than pristine AlNNT (5,5) due to CO_2_ adsorption. The molecular orbital analysis showed that the electronic interaction is primarily between the CO_2_ molecule and (5,5) AlNNT, as the HOMO was only on the (5,5) AlNNT, and the LUMO was on both the (5,5) AlNNT and CO_2_, as seen in Figure 3D. The charge density diagram in Figure 3C demonstrates that the CO_2_ molecule interacts with the (5,5) AlNNT through charge transfer, as the charge density is distributed over both the (5,5) AlNNT and CO_2_. Moreover, there was a decrease in the Fermi energy from −0.180 eV to −0.184 eV, as shown in Figure 3B.

### 2.4. CO_2_ Adsorbed on Si-(5,5) AlNNT [Si-(5,5) AlNNT/CO_2_]

**Figure 4:**
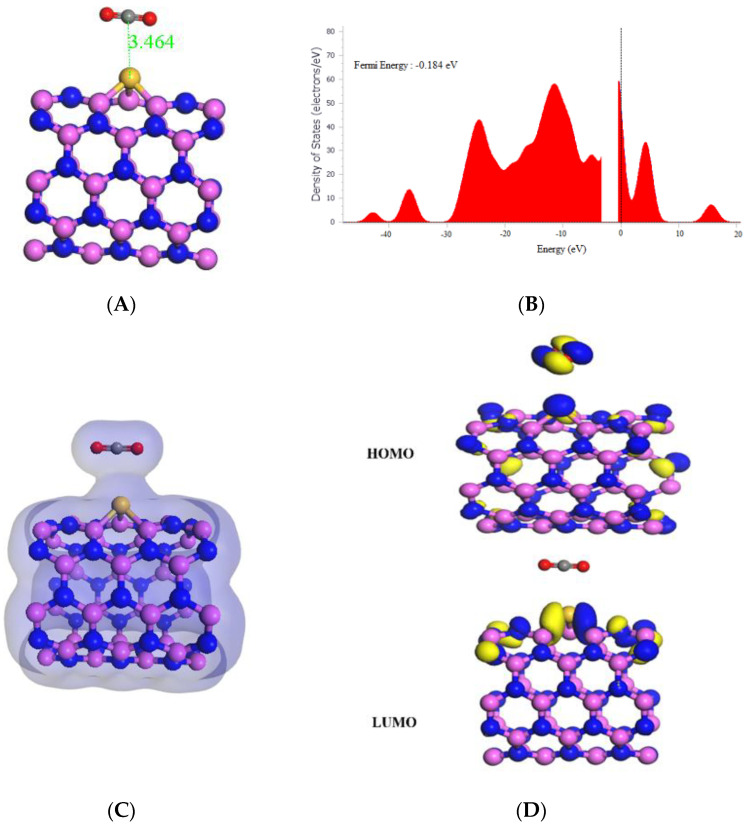
(**A**) Geometry optimized structure with the adsorption distance measured in Å, (**B**) density of states, (**C**) charge density, and (**D**) frontier HOMO and LUMO orbitals for the Si-(5,5) AlNNT and its interaction with CO_2_.

As seen in Table 1, the interaction between CO_2_ and Si-(5,5) AlNNT was identified as a strong adsorption process with an adsorption energy of −19.69 kcal/mol. The observed decrease in the band gap (Eg) from 3.725 eV to 3.926 eV following CO_2_ adsorption suggests that the doping process introduced new electronic states in Si-(5,5) AlNNT, as depicted in Figure 4B. This could potentially impact its electrical conductivity and optical properties. The decrease in Eg further implies that Si-(5,5) AlNNT might have higher conductivity after CO_2_ adsorption. The sensing potential of 5.40% reported in Table 1 suggests that Si-(5,5) AlNNT could be a promising candidate for CO_2_ sensing applications with a relatively short recovery time of 178.689 s, which is approximately 2.978 min. The results in Table 1 also further reveal that the chemical potential (µ) of −4.117 eV, global hardness (η) of 1.963 eV, and electrophilicity index (ω) of 4.317 eV indicate that Si-(5,5) AlNNT could have become more susceptible to chemical reactions after CO_2_ adsorption. Figure 4D shows that the HOMO was on both the Si-(5,5) AlNNT and CO_2_, while the LUMO was present solely on the AlNNT (5,5). Moreover, the charge density diagram in Figure 4C demonstrates that the charge density has covered both the Si-(5,5) AlNNT and CO_2_, suggesting a strong interaction between them.

### 2.5. CO Adsorbed on (5,5) AlNNT [(5,5) AlNNT/CO]

**Figure 5:**
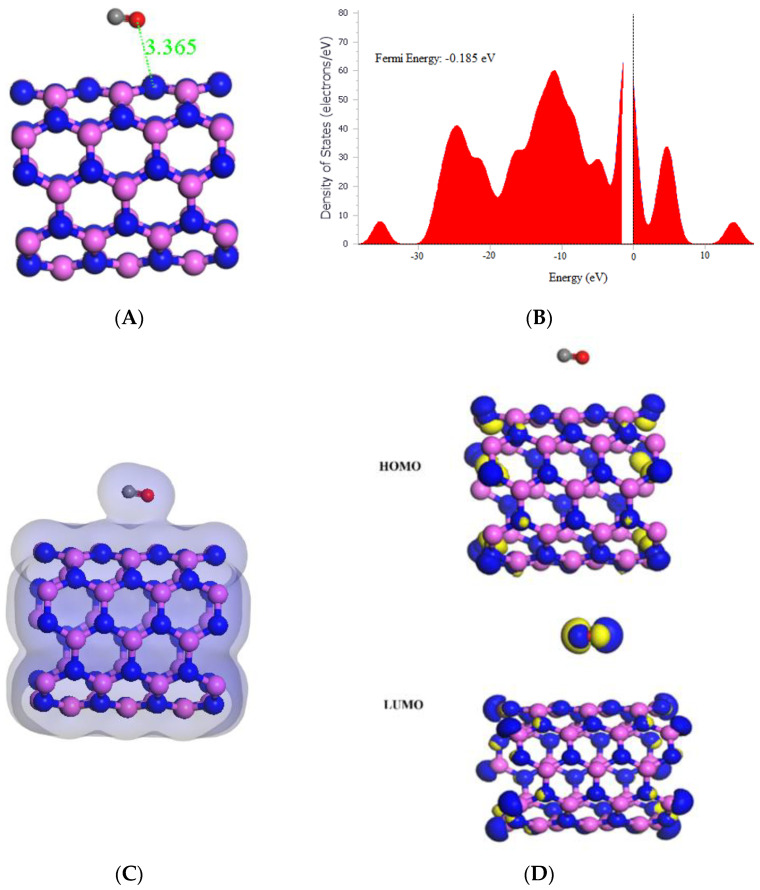
(**A**) Geometry optimized structure with the adsorption distance measured in Å, (**B**) density of states, (**C**) charge density, and (**D**) frontier HOMO and LUMO orbitals for the (5,5) AlNNT and its interaction with CO.

The findings on the interaction of carbon monoxide (CO) with (5,5) AlNNT through adsorption are presented in Table 1. This study revealed a strong binding between the CO molecule and the (5,5) AlNNT surface, with a significant adsorption energy of −25.20 kcal/mol. This interaction caused a significant change in the electronic properties of (5,5) AlNNT, resulting in a reduction in the band gap from 4.090 eV to 3.718 eV. The results indicate that (5,5) AlNNT has the potential to be used as a CO sensor, with a calculated sensing potential of 9.095%. However, the relatively long recovery time of 1,739,274.942 s, which is approximately 20.130 days, indicates that further improvements may be necessary to optimize sensor performance. The charge density diagram in Figure 5C suggests the formation of a hybrid state between the (5,5) AlNNT surface and the CO molecule. Besides, Table 1 shows changes in the chemical potential, global hardness, and electrophilicity index of (5,5) AlNNT after CO adsorption. The chemical potential slightly shifts from −4.265 eV to −4.410 eV, indicating a destabilization of the system upon adsorption. The global hardness decreases from 2.045 eV to 1.859 eV, indicating a decrease in resistance to deformation. Lastly, the electrophilicity index increases from 4.447 eV to 5.230 eV, indicating that the system becomes more electron-seeking. Additionally, the molecular orbital analysis in Figure 5D reveals that the LUMO was localized only on the (5,5) AlNNT, while the HOMO was present on both (5,5) AlNNT and CO.

### 2.6. CO Adsorbed on Si-(5,5) AlNNT [Si-(5,5) AlNNT/CO]

**Figure 6:**
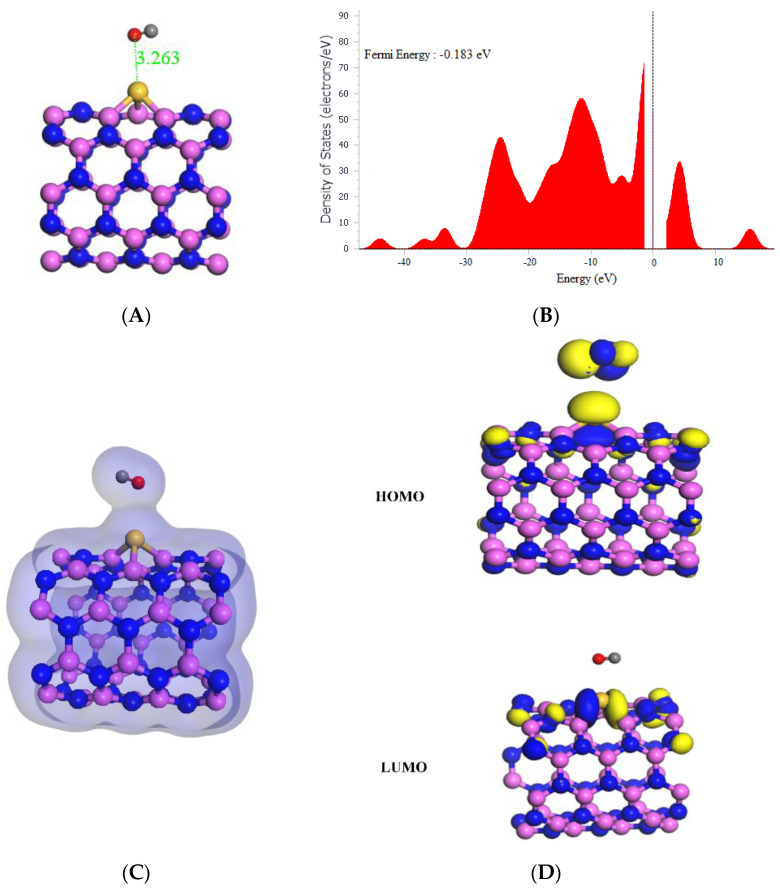
(**A**) Geometry optimized structure with the adsorption distance measured in Å, (**B**) density of states, (**C**) charge density, and (**D**) frontier HOMO and LUMO orbitals for the Si-(5,5) AlNNT and its interaction with CO.

As shown in Table 1, the adsorption of CO onto Si-(5,5) AlNNT resulted in a strong and favorable interaction, with a high adsorption energy of −20.78 kcal/mol. The HOMO and LUMO values of the system were −6.033 eV and −2.202 eV, respectively, with a band gap of 3.831 eV. The slight change in Fermi energy from −0.193 eV to −0.183 eV after adsorption was observed in Figure 6B. The Si-(5,5) AlNNT showed a sensing potential of 4.29%, suggesting it could be a potential candidate for CO sensing applications, with a relatively short recovery time of 1099.171 s, which is approximately 18.320 min, which would support its real-time application. After adsorption, the chemical potential (µ) decreased from −4.131 eV to −4.118 eV, indicating new chemical states were introduced in the Si-(5,5) AlNNT. The global hardness (η) decreased from 1.863 eV to 1.916 eV, implying that the Si-AlNNT (5,5) became less resistant to deformation after adsorption. On the other hand, the electrophilicity index (ω) increased from 4.425 eV to 4.580 eV, suggesting that the Si-(5,5) AlNNT may have become more susceptible to chemical reactions after adsorption. The molecular orbital analysis in Figure 6D indicated that the LUMO was localized only on the Si-(5,5) AlNNT, while the HOMO was on both the Si-(5,5) AlNNT and CO. The charge density diagram in Figure 6C suggested that there was a charge transfer between the Si-(5,5) AlNNT and CO, with the charge density distributed on both the nanotube and CO. Thus, this charge transfer may be responsible for the strong adsorption energy observed in the system.

### 2.7. Potential Challenges of Implementing AlNNT-Based Sensors

AlNNT-based sensors hold significant promise for real-world applications due to their distinctive properties; however, their practical implementation presents notable challenges and constraints. To begin with, the synthesis and production of AlNNTs can be intricate and costly, raising concerns about purity and scalability [44,57]. Moreover, these nanotubes demonstrate sensitivity to environmental factors like temperature and humidity, potentially impacting their performance and long-term reliability [40]. Consequently, it is imperative to focus on optimizing production parameters and mastering control over nanotube growth.

Lastly, when comparing the (5,5) nanotube configuration to zig-zag and chiral configurations, the (5,5) configuration exhibited the greatest stability following molecular adsorption [58]. Given our study context, we may anticipate distinct behaviors for MWNTs, zig-zag, and chiral AlNNT configurations when exposed to CO and CO_2_ molecules. For the other configurations, we recommend both computational and experimental assessments in future research endeavors.

## 3. Materials and Methods

### Computational Details

The Nanotube builder software [54] and Avogadro software [56,59,60] were utilized to create models of the armchair AlNNT (5,5), Si-doped AlNNT (5,5), Carbon monoxide (CO), and Carbon dioxide (CO_2_) gas molecules for the study, where AlNNT (5,5) and Si-doped AlNNT (5,5) are the pristine aluminum nitride nanotube and silicon replacing a nitrogen atom in the aluminum nitride nanotube, respectively. Subsequently, the molecular geometries were optimized using DFT and Quantum Espresso software [61]. The quantum chemical periodic approach was used throughout the calculations. We employed a single starting geometry per system for the adsorption studies. Equation (1) presents the adsorption model in the following form:

***E*_*ads*_** = E_adsorbate_adsorbent_ − (E_adsorbate_ + E_adsorbent_) (1)

In this equation, E_ads_ represents the adsorption energy, E_adsorbate_adsorbent_ denotes the cluster energy, E_adsorbate_ represents the ground state energy of the relaxed adsorbate molecule, and E_adsorbent_ refers to the energy of the relaxed adsorbent molecule being investigated.

The recovery time (RT) plays a crucial role in the development of sensors, as it is influenced by the interaction strength, which in turn determines the difficulty of the adsorption process. A more negative value of Eads results in a higher recovery time (RT).

(2) $$\tau ={v0}^{-1}\exp\left(\frac{{-E}_{ads}}{kT}\right)$$

where τ = recovery time; ν0 = attempt frequency; k = Boltzmann’s constant (∼2.0 × 10^−3^ kcal/mol. K); and T = temperature. According to experimental research studies, different photonic frequencies (υ0), thermal energy, and irradiation can be employed for the desorption of molecules [62,63]. In this study, υ0 = 10^12^ s^−1^ and T = 300 K will be the values for the attempt frequency and temperature, respectively, throughout the research. The choice of υ0 = 10^12^ s^−1^ and T = 300 K for attempt frequency and temperature in this study is justified by their consistent usage in the work of Wang et al. (2022) [61].

The calculation of the band gap (Eg) between the highest occupied molecular orbital (HOMO) and the lowest unoccupied molecular orbital (LUMO) will be performed using the following Equation (3):

(3) $$E_{g}=E_{LUMO}-E_{HOMO}$$

where the energy of the LUMO and the HOMO are denoted by E_LUMO_ and E_HOMO_, respectively. 

To evaluate the sensitivity of the nanomaterials, the variation in the energy gap (E_g_) will be determined using the following equation, where E_g1_ represents the energy gap of the aluminum nitride nanotube, and E_g2_ represents the energy gap of the nanotube-adsorbate complex:

(4) $$∆Eg=[(E_{g2}-E_{g1})/E_{g1}]\times 100$$

The descriptors of chemical potential (µ), global hardness (η), and electrophilicity index (ω) offer valuable insights into the chemical reactivity and stability of interactions. They provide a means to understand the energy stabilization that occurs when a molecular system undergoes charge transfer from its surrounding environment. The electrophilicity index (ω) is closely related to both the chemical potential (µ) and global hardness (η), and their relationship can be expressed as follows:

***ω*** **=** ***µ*^2^**/**2*η*** (5)

The global hardness parameter provides an indication of the molecular system’s ability to resist changes in electron distribution. It is calculated by taking half of the difference between the energies of the highest occupied molecular orbital (HOMO) and the lowest unoccupied molecular orbital (LUMO). The chemical potential (µ) is determined by averaging the energies of the HOMO and LUMO. These calculations were performed using the computational resources available at the South African Centre of High-Performance Computing (CHPC) cluster.

## 4. Conclusions

This computational research focused on investigating the interactions between carbon dioxide (CO_2_) and carbon monoxide (CO) gas molecules with pristine (5,5) AlNNT and silicon-doped (5,5) AlNNT. The adsorption of CO_2_ on (5,5) AlNNT showed a strong binding with significant adsorption energy, indicating its potential as a CO_2_ sensor. However, the slow recovery time after CO_2_ adsorption may limit its practical application. The electronic structure of (5,5) AlNNT was modified by CO_2_ adsorption, leading to a decrease in the band gap and changes in the HOMO and LUMO values. Similarly, the adsorption of CO on (5,5) AlNNT exhibited a strong binding, suggesting its potential as a CO sensor. However, the relatively long recovery time indicates the need for further optimization. The interaction between CO_2_ and Si-(5,5) AlNNT demonstrated a strong adsorption process, with changes in the band gap and the introduction of new electronic states. Si-(5,5) AlNNT showed promising potential as a CO_2_ sensor with a relatively short recovery time. Additionally, the interactions between CO and Si-(5,5) AlNNT resulted in strong and favorable adsorption, with changes in the chemical potential, global hardness, and electrophilicity index. These findings suggest that Si-(5,5) AlNNT could be a potential candidate for CO sensing applications. Molecular orbital analysis and charge density diagrams provided insights into the electronic interaction and charge transfer between the nanotubes and the molecules. Overall, this research sheds light on the potential applications of pristine and silicon-doped aluminum nitride nanotubes in gas sensing and provides valuable insights into their electronic properties and interactions with CO_2_ and CO molecules.

## Figures and Tables

**Table 1:** Computed adsorption energy (Eads) measured in kcal/mol, HOMO and LUMO energies in eV, band gap (Eg) in eV, chemical potential (µ) in eV, global hardness (η) in eV, electrophilicity index (ω) in eV, Recovery time (τ) in Seconds and sensing potential (%Eg) in % for the nanotubes and nanotube/gas complexes.

Model	Eads	HOMO	LUMO	Eg	%Eg	τ	µ	η	ω
(5,5) AlNNT	-	−6.310	−2.220	4.090	-	-	−4.265	2.045	4.447
Si-(5,5) AlNNT	-	−5.988	−2.273	3.725	-	-	−4.131	1.863	4.580
(5,5)AlNNT/ CO_2_	−24.36	−6.026	−2.302	3.724	8.95	428,899.920	−4.164	1.862	4.656
Si-(5,5) AlNNT/CO_2_	−19.69	−6.080	−2.154	3.926	5.40	178.689	−4.117	1.963	4.317
(5,5) AlNNT/CO	−25.20	−6.000	−2.282	3.718	9.095	1,739,274.942	−4.410	1.859	5.230
Si-(5,5) AlNNT/CO	−20.78	−6.033	−2.202	3.831	4.29	1099.171	−4.118	1.916	4.425

## Data Availability

All data for this work in contained in the article.
